# Supplementary material for: Ezrin Contributes to the Plasma Membrane Expression of PD–L1 in A2780 Cells
Source: J Clin Med. 2022 Apr 27;11(9):2457. doi: 10.3390/jcm11092457 (PMC9100183; doi:10.3390/jcm11092457)
Supplement: Supplementary file 1 [file jcm-11-02457-s001.zip › jcm-1672769-SI.pdf]

Journal of
Clinical Medicine

MDPI

Supplementary Materials

Ezrin Contributes to the Plasma Membrane Expression of PD–L1 in A2780 Cells

Mayuka Tameishi [1,†], Honami Ishikawa [1,†], Chihiro Tanaka [1], Takuro Kobori [1], Yoko Urashima [1], Takuya Ito [2], and Tokio Obata [1,*]

[1] Laboratory of Clinical Pharmaceutics, Faculty of Pharmacy, Osaka Ohtani University, 584–8540 Tondabayashi, Japan; u4117083@osaka-ohtani.ac.jp (M.T.); u4118007@osaka-ohtani.ac.jp (H.I.); u4117078@osaka-ohtani.ac.jp (C.T.); koboritaku@osaka-ohtani.ac.jp (T.K.); urasiyo@osaka-ohtani.ac.jp (Y.U.)
[2] Laboratory of Natural Medicines, Faculty of Pharmacy, Osaka Ohtani University, 584–8540 Tondabayashi, Japan; itoutaku@osaka-ohtani.ac.jp
* Correspondence: obatatoki@osaka-ohtani.ac.jp; Tel.: +81-721-24-9371
† These authors contributed equally to this work.

Whole western blots to confirm the reactivity of all antibodies used in this study

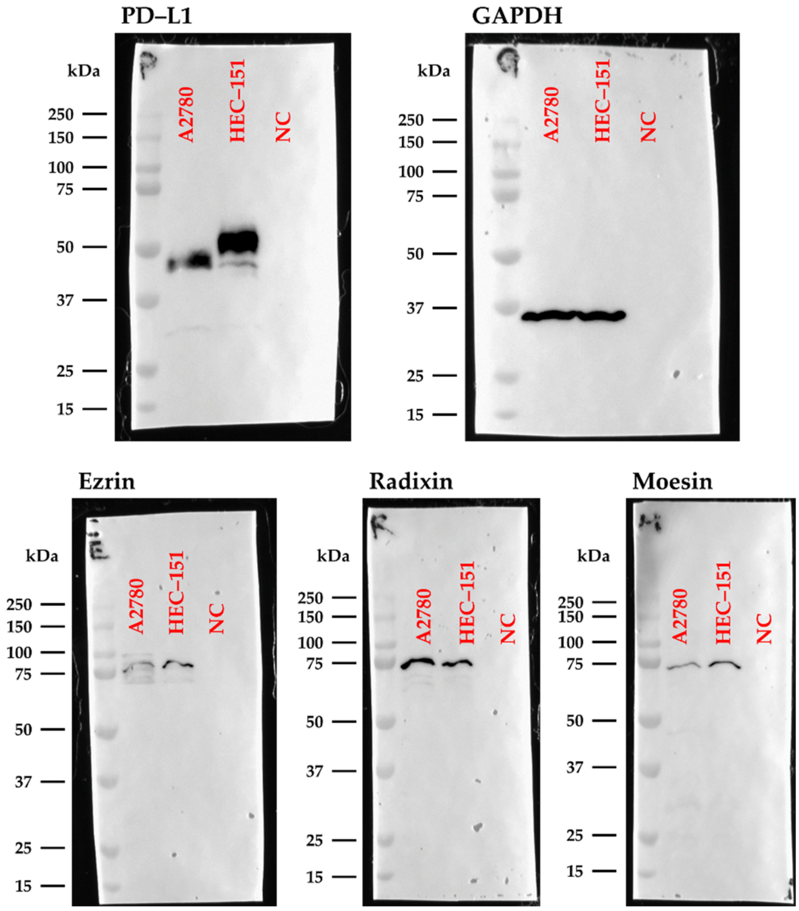

Figure S1. Whole western blots to confirm the reactivity of all antibodies used in this study. The original western blotting membrane to detect the protein expression of programmed death ligand–1 (PD–L1), ezrin, radixin, and moesin as well as the corresponding glyceraldehyde–3–phosphate dehydrogenase (GAPDH). Blots are labeled according to the corresponding target protein. Each blot in left lane; Whole cell lysates of A2780 cells, middle lane; Whole cell lysates of HEC–151 cells (positive control), right lane; Negative control (NC, no protein). Molecular weights are indicated in kDa.

Original source images for all data obtained by immunoblots

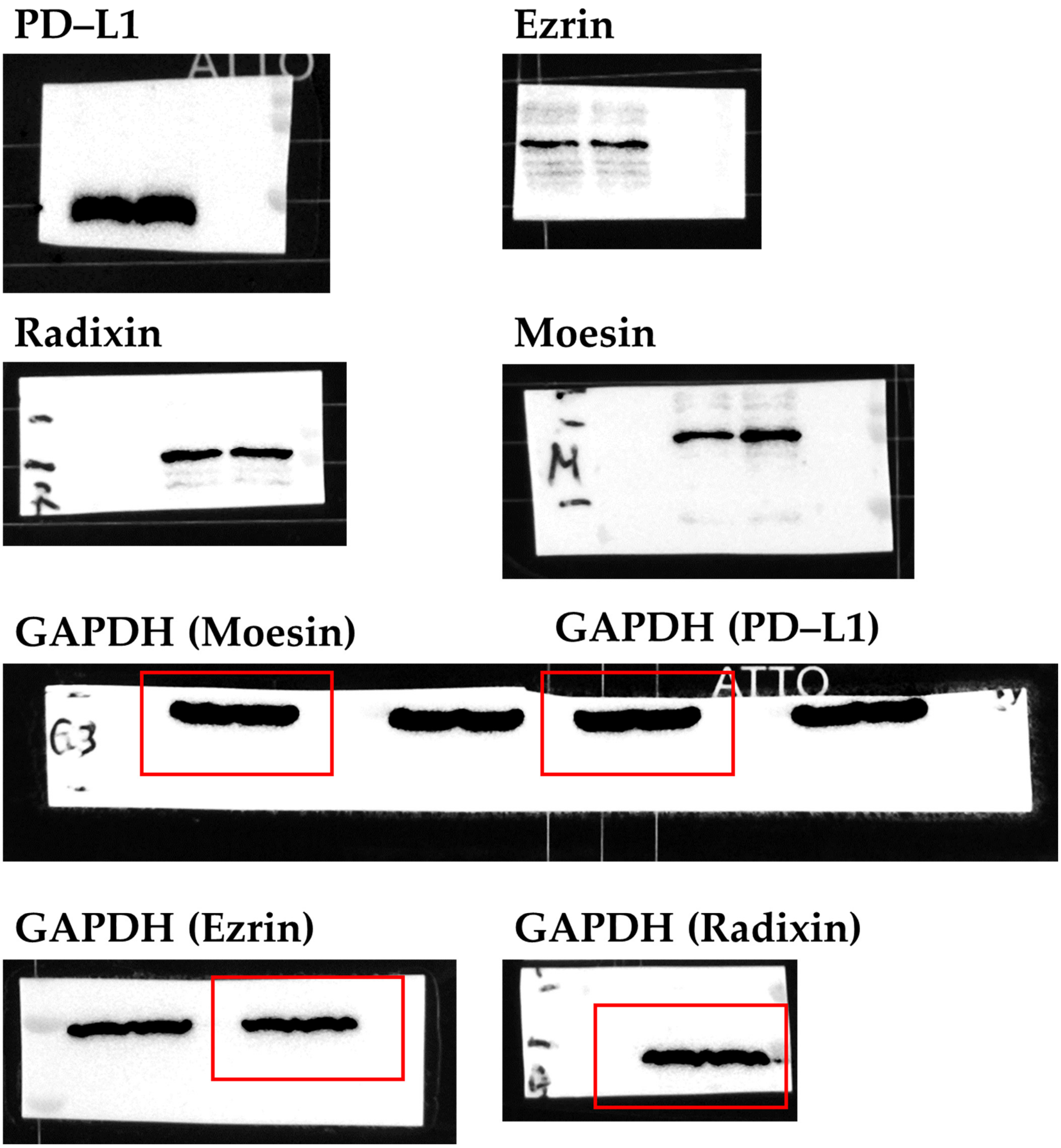

Figure S2. Original source images for all data obtained by immunoblots. The original western blotting membrane to detect the protein expression of programmed death ligand–1 (PD–L1), ezrin, radixin, and moesin as well as the corresponding glyceraldehyde–3–phosphate dehydrogenase (GAPDH) used as a loading control shown in Figure 1b.

Negative fluorescence staining for A2780 cells in confocal laser scanning microscopy analysis

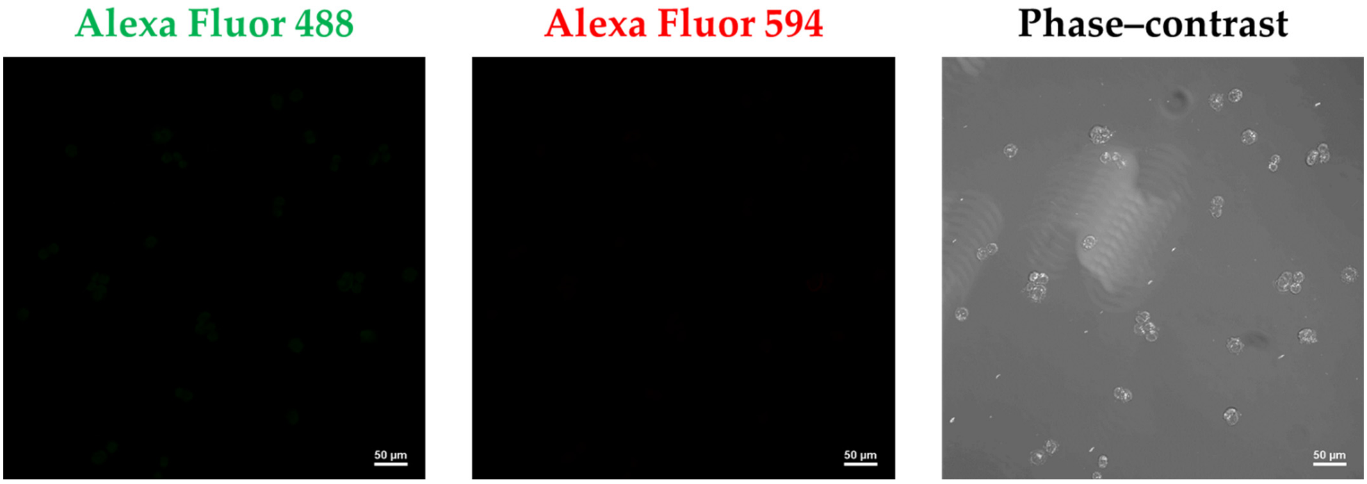

Figure S3. Negative fluorescence staining for A2780 cells in confocal laser scanning microscopy analysis. Negative fluorescence staining in Figure 3. Left and middle panels; Fluorescence images of goat anti–rabbit IgG (heavy + light) secondary antibodies conjugated with an Alexa Fluor 488 or an Alexa Fluor 594, respectively, without primary antibodies against PD–L1 or each ERM, right panel; Phase–contrast image. Scale bars: 50 µm. All images were captured by confocal laser scanning microscopy.

Influence of siRNAs against each target gene on the cell viability of A2780 cells

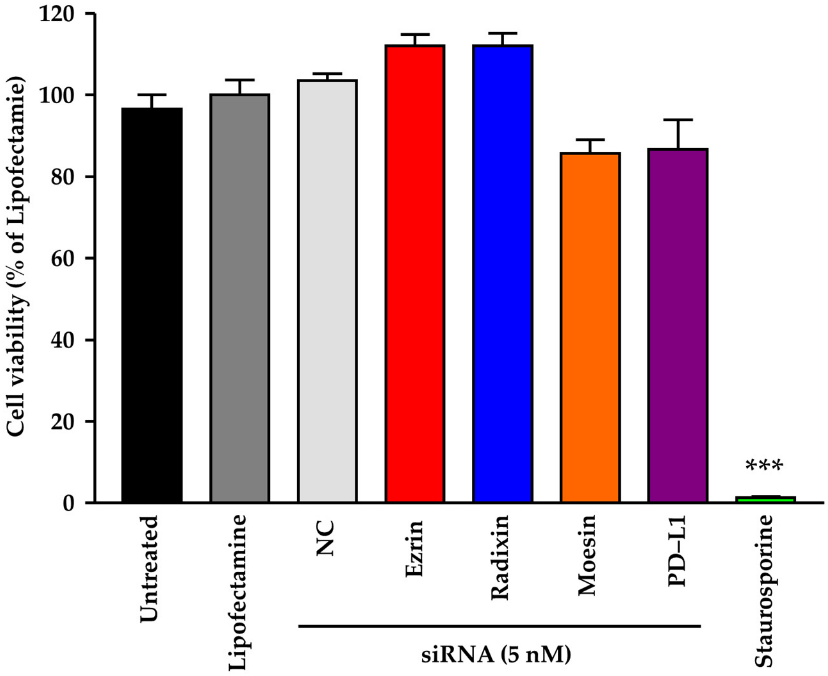

Figure S4. Influence of siRNAs against each target gene on the cell viability of A2780 cells. Cells were treated with the transfection medium (Untreated), transfection reagent (Lipofectamine), nontargeting control (NC) small interfering (si) RNA, and specific siRNAs for ezrin, radixin, moesin, or programmed death ligand–1 (PD–L1) at the concentrations of 5 nM, and then cultured for 4 days. Cell viability of A2780 cells was assessed with the PrestoBlue cell viability reagent. Staurosporine 1.0 μM is included as a positive control to reduce *in vitro* cell viability. $n = 6$, ***$p < 0.001$ vs. Lipofectamine. All data were expressed as the mean ± SEM and analyzed by one–way ANOVA followed by Dunnett's test.

Materials and Methods for Figure S4

Cell Viability Assay

A2780 cells at a density of 5.0×10^3 cells were cultured in 96–well cell culture plates (Corning, Glendale, AZ, USA) overnight at 37 °C in a humidified atmosphere with 5% CO_2 to allow for attachment. Then, cells were treated with siRNAs as described in the Main Manuscript and 1.0 μM of staurosporine (Merck, Darmstadt, Germany), a suitable positive control to damage *in vitro* cell viability, for 4 days without exchanging medium. Thereafter, cells were incubated with a PrestoBlue Cell Viability Reagent (Thermo Fisher Scientific, Tokyo, Japan), a fast and sensitive assay for assessing cell viability [1,2], at 37 °C for 10 min under humidified conditions with 5% CO_2, protected from direct light. After that, fluorescence signals were detected at wavelengths of 560 nm (excitation) and 590 nm (emission) using a Synergy HTX Multi–Mode Microplate Reader (Bio Tek Instrument, Winooski, VT, USA).

References for Figure S4

1. Lall, N.; Henley–Smith, C.J.; De Canha, M.N.; Oosthuizen, C.B.; Berrington, D. Viability Reagent, PrestoBlue, in Comparison with Other Available Reagents, Utilized in Cytotoxicity and Antimicrobial Assays. *Int. J. Microbiol.* **2013**, *2013*, 420601, doi:10.1155/2013/420601.
2. Boncler, M.; Rozalski, M.; Krajewska, U.; Podsedek, A.; Watala, C. Comparison of PrestoBlue and MTT assays of cellular viability in the assessment of anti–proliferative effects of plant extracts on human endothelial cells. *J. Pharmacol. Toxicol. Methods* **2014**, *69*, 9–16, doi:10.1016/j.vascn.2013.09.003.

Changes in the mRNA expression levels of interferon (IFN)–ɤ by gene silencing of ezrin, radixin, and moesin in A2780 cells

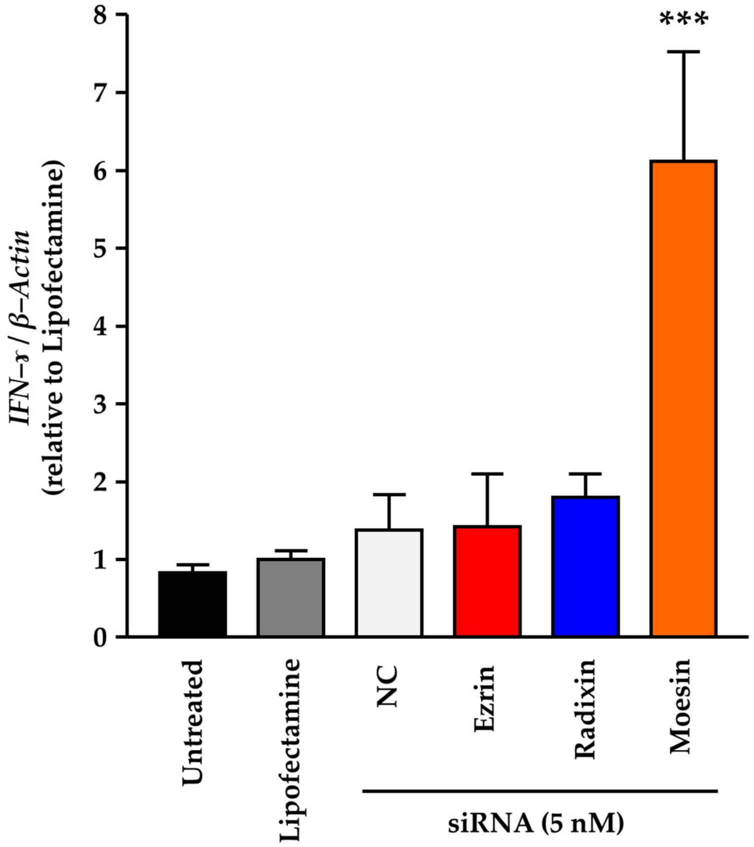

Figure S5. Changes in the mRNA expression level of interferon (IFN)–ɤ by gene silencing of ezrin, radixin, and moesin in A2780 cells. Cells were incubated with the transfection medium (Untreated), transfection reagent (Lipofectamine), nontargeting control (NC) small interfering (si) RNA, and specific siRNAs for ezrin, radixin, or moesin and then cultured for 4 days. Gene expression level of IFN–ɤ mRNA normalized with β–Actin in cells treated with each siRNA relative to that in cells treated with the transfection reagent alone. $n = 3$, ***$p < 0.001$ vs. Lipofectamine. All data were expressed as the mean ± SEM and analyzed by one–way ANOVA followed by Dunnett's test.

Table S1. List of primer sequences

Primers	Primer Sequence (5′→3′)
h–*β–Actin* (forward)	TGGCACCCAGCACAATGAA
h–*β–Actin* (reverse)	CTAAGTCATAGTCCGCCTAGAAGCA
h–*PD–L1* (forward)	CAATGTGACCAGCACACTGAGAA
h–*PD–L1* (reverse)	GGCATAATAAGATGGCTCCCAGAA
h–*Ezrin* (forward)	ACCATGGATGCAGAGCTGGAG
h–*Ezrin* (reverse)	CATAGTGGAGGCCAAAGTACCACA
h–*Radixin* (forward)	GAATTTGCCATTCAGCCCAATA
h–*Radixin* (reverse)	GCCATGTAGAATAACCTTTGCTGTC
h–*Moesin* (forward)	CCGAATCCAAGCCGTGTGTA
h–*Moesin* (reverse)	GGCAAACTCCAGCTCTGCATC
h–*IFN–ɤ* (forward)	CTTTAAAGATGACCAGAGCATCCAA
h–*IFN–ɤ* (reverse)	GGCGACAGTTCAGCCATCAC
h–*TNF* (forward)	ACAACCCTCAGACGCCACAT
h–*TNF* (reverse)	GTGGAGCCGTGGGTCAGTAT
h–*IL–6* (forward)	AAGCCAGAGCTGTGCAGATGAGTA
h–*IL–6* (reverse)	TGTCCTGCAGCCACTGGTTC

Table S2. List of antibodies

Antibodies	Manufacturer	Cat. No.	Dilution	Lot No.
rabbit anti–ezrin	Cell Signaling Technology	3145	1:2,000 (WB)<br>1:50 (IF)	3
rabbit anti–radixin	Gene Tex	GTX105408	1:2,000 (WB)<br>1:100 (IF)	39939
rabbit anti–moesin	Cell Signaling Technology	3150	1:2,000 (WB)<br>1:50 (IF)	2
Alexa Fluor 488–conjugated rabbit anti–PD–L1	Cell Signaling Technology	25048	1:50 (IF)	2
Alexa Fluor 488–conjugated goat anti–rabbit IgG	Thermo Fisher Scientific	R37116	1:25 (IF)	2277676
Alexa Fluor 594–conjugated goat anti–rabbit IgG	Thermo Fisher Scientific	R37117	1:25 (IF)	2237275
HRP–conjugated rabbit anti–PD–L1	Cell Signaling Technology	51296s	1:1,000 (WB)	5
mouse anti–GAPDH	Merck	MAB374	1:20,000 (WB)	3432602
HRP–conjugated anti–rabbit IgG (heavy + light)	SeraCare Life Sciences	5220–0336	1:10,000 (WB)	10468833
HRP–conjugated anti–mouse IgG (heavy + light)	SeraCare Life Sciences	5220–0341	1:10,000 (WB)	10387619
APC–conjugated mouse anti–human PD–L1	BioLegend	329708	4.0 µg/test (FC)	B311349

western blotting; WB, immunofluorescence; IF, flow cytometry; FC, horseradish peroxidase; HRP, GAPDH; glyceraldehyde–3–phosphate dehydrogenase allophycocyanin; APC
